# Emerging Epidemiological Data on Rare Intellectual Disability Syndromes from Analyzing the Data of a Large Iranian Cohort

**DOI:** 10.34172/aim.2023.29

**Published:** 2023-04-01

**Authors:** Farzane Zare Ashrafi, Tara Akhtarkhavari, Zohreh Fattahi, Maryam Asadnezhad, Maryam Beheshtian, Sanaz Arzhangi, Hossein Najmabadi, Kimia Kahrizi

**Affiliations:** ^1^Genetics Research Center, University of Social Welfare and Rehabilitation Sciences, Tehran, Iran

**Keywords:** Consanguinity, Epidemiology, Intellectual disability, Iran, Rare diseases

## Abstract

**Background:**

Intellectual disability (ID) is a genetically heterogeneous condition, and so far, 1679 human genes have been identified for this phenotype. Countries with a high rate of parental consanguinity, such as Iran, provide an excellent opportunity to identify the remaining novel ID genes, especially those with an autosomal recessive (AR) mode of inheritance. This study aimed to investigate the most prevalent ID genes identified via next-generation sequencing (NGS) in a large ID cohort at the Genetics Research Center (GRC) of the University of Social Welfare and Rehabilitation Sciences.

**Methods:**

First, we surveyed the epidemiological data of 619 of 1295 families in our ID cohort, who referred to the Genetics Research Center from all over the country between 2004 and 2021 for genetic investigation via the NGS pipeline. We then compared our data with those of several prominent studies conducted in consanguineous countries. Data analysis, including cohort data extraction, categorization, and comparison, was performed using the R program version 4.1.2.

**Results:**

We categorized the most common ID genes that were mutated in more than two families into 17 categories. The most common syndromic ID in our cohort was AP4 deficiency syndrome, and the most common non-syndromic autosomal recessive intellectual disability (ARID) gene was *ASPM*. We identified two unrelated families for the 36 ID genes. We found 14 genes in common between our cohort and the Arab and Pakistani groups, of which three genes (*AP4M1*, *AP4S1*, and *ADGRG1*) were repeated more than once.

**Conclusion:**

To date, there has been no comprehensive targeted NGS platform for the detection of ID genes in our country. Due to the large sample size of our study, our data may provide the initial step toward designing an indigenously targeted NGS platform for the diagnosis of ID, especially common ARID in our population.

## Introduction

 Intellectual disability (ID) is a frequent neurodevelopmental disorder diagnosed with cognitive and adaptive deficits before the age of 18 years.^1^ ID is estimated to affect 1%–3% of the global population. It can manifest as an isolated clinical manifestation or as a syndromic phenotype, as well as other physical and mental abnormalities such as behavioral problems. Based on etiology, ID can happen due to both genetic factors and pre-and post-natal environmental factors.^2^ Genetic factors contribute to a significant number of ID cases, and studies show that the most severe and profound ID patients are affected by monogenic disorders.^2,3^ Based on SysNDD (a database that contains a catalogue of published genes implicated in neurodevelopmental disorders; last update: 6/25/2022), out of 1679 genes involved in ID, 982 show an autosomal recessive (AR) mode of inheritance, 527 exhibit autosomal dominant (AD) inheritance, 154 genes show X-linked inheritance, and others are involved in ID through mitochondrial inheritance and somatic mutations.^4^ Prior to the advent of next-generation sequencing (NGS), the diagnosis of monogenic ID was not sufficiently fast and efficient. However, with the introduction of this technology, the identification of disease-causing variants in monogenic cases of ID has improved drastically.^5^ Moreover, epidemiological studies of ID in inbred countries can provide reliable data about the most prevalent ID genes or gene groups. As shown in SysNDD, autosomal recessive intellectual disability (ARID) is one of the important forms of monogenic IDs. This form of ID is a clinically and genetically extremely heterogeneous condition and has major contribution to the etiology of ID.^6^ It is estimated that in outbred countries, ARID accounts for about 10% of all diagnosed ID cases and contributes to 15–20% of all undiagnosed patients.^6,7^ At the same time, in countries with a high rate of parental consanguinity, the incidence of ARID shows a three-to four-fold increase, and rare ARIDs are more common among these populations.^1,6^ Although a large number of ARID genes have been identified, the abundance of these genes remains unrecognized, and there is no extensive targeted NGS platform for diagnosing ARIDs with a high confidence rate.^6^ Countries with a high rate of parental consanguinity provide an excellent opportunity for identification of the remaining novel genes involved in ARIDs. Since Iran is a Middle Eastern country with a parental consanguinity rate of approximately 40%, it provides a suitable population reservoir for the epidemiological study of IDs, especially ARIDs.^1^ The main goal of this study was to investigate the prevalence of genes identified using NGS in a large ID cohort at the Genetics Research Center of the University of Social Welfare and Rehabilitation Sciences. To the best of our knowledge, there is no comprehensive targeted NGS platform to detect ID genes in our country; therefore, considering the large sample size of this cohort, the present study may be the first step towards the design of an NGS platform for the diagnosis of ID in our country. We also compared the results of our study with those of several similar studies from other groups in consanguineous families originating from the Middle East to investigate overlapping gene defects with neighboring countries.

## Materials and Methods

 The epidemiological data obtained for this study were extracted from unpublished data and articles previously published by our research team.^1,8-10^ In order to develop the cohort, we established a genetic counseling network from all 31 provinces of Iran to include all ethnic groups in our country. Iranian families were referred by physicians or clinical geneticists from all over the country.^11^ The above-mentioned cohort consisted of a total of 1295 Iranian families who were referred to the Genetics Research Center of the University of Social Welfare and Rehabilitation Sciences (Iran) between 2004 and 2021 to identify genetic causes of ID. We performed total population sampling on our Iranian ID cohort. We defined the exclusion criteria as follows: families with chromosomal abnormalities, families with Fragile X syndrome, and inconsistent families. In 2011, our team studied 136 consanguineous families and applied homozygosity mapping, exon enrichment and targeted next generation sequencing.^9^ In another study, we performed whole-genome sequencing and/or whole exome-sequencing on 404 consanguineous families;^1^ it should be mentioned that these families also included undiagnosed families from our previous study. In 2019, we applied whole exome-sequencing to 100 sporadic ID cases.^8^ We also added ID families from the unpublished data. In total, we had 619 Iranian families with ID with definitive diagnoses of the genetic causes of this disorder. To identify the most prevalent genes in our cohort, data extraction was performed using the R program version 4.1.2.

 We also compared the most prevalent genes with multiple papers that published their ID cohorts. Since Iran has a high consanguinity rate, we chose papers from countries with high rates of consanguinity. These include papers from Pakistan and the Arabs of West Asia and North Africa.^12-18^ Table S1 lists the genes used for the comparison. In the comparison of genes among the three groups, the following items were excluded.

1. Families with copy number variations
2. Families with multiple candidate genes
3. Samples that were investigated by a method other than NGS

We should mention that in this study, we did not have any information about ethnicity groups in other ID cohort papers, so we could not compare our data of ethnicity groups with the same ethnicity in neighboring countries.

## Results

**Figure 1:**
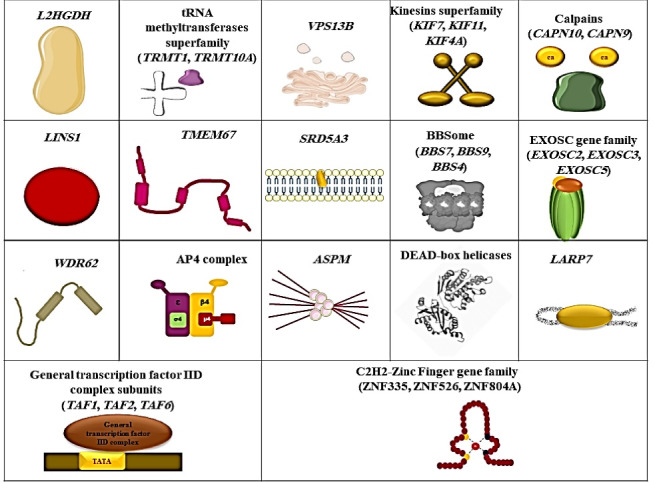
The Most Commonly Reported Genes or Gene Groups in our Cohort.

**Figure 2:**
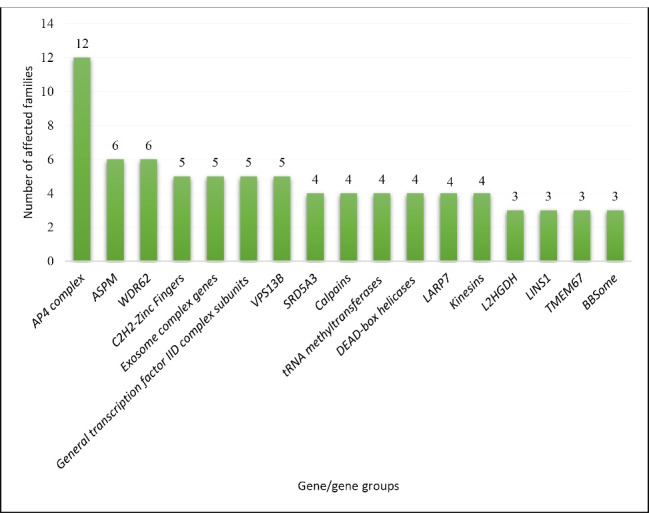
Number of Families with the Most Common Mutated Genes or Gene Groups in Our Cohort.

 Out of 619 of the 1295 families in our ID cohort, we found 56 families that were reported twice in our cohort (56 families with mutations in 36 genes) and 65 families with a gene that was reported at least three times within the cohort (65 families with mutations in 17 genes). Based on the function of the genes, we categorized our most common genes, as depicted in Figure 1, and the number of families with mutations in each category is shown in Figure 2.

 Further detailed data regarding the putative function of each gene in the pathogenesis of ID and related phenotypes of each gene/gene group are presented in Table 1 and Table 2. Furthermore, for multiple genes, we found two unrelated affected families, as listed in Table 3.

**Table 1:** Functions of the Genes and their Associated Phenotypes

**Category**	**Function of the Genes and Implicated Phenotypes**
Adaptor-related protein complex 4 (AP4)	The AP4 complex is one of the five members of the Adapter Protein family, which is involved in the post-Golgi pathways in transporting cargo from the trans-Golgi to endosomes and autophagosomal structures.^19^ This complex consists of four subunits, encoded by *AP4B1*, *AP4E1*, *AP4M1*, and *AP4S1*. The AP4 complex could be involved in the transportation of various cargoes, including low-density lipoprotein receptor, amyloid precursor protein, α-amino-3-hydroxy-5-methyl-4-isoxazolepropionic acid receptors, ATG9A, and δ2 glutamate receptors.^20^ All which are essential for the proper functioning of the brain.^21-26^ Mutations in AP4 complex genes cause AP4 deficiency syndrome, which is characterized by intellectual disability, spastic tetraplegia, developmental delay, speech disorder, microcephaly, and inability to walk.^19^
Abnormal spindle-like, microcephaly-associated; *ASPM*	*ASPM *encodes ASPM, a protein localized at the centrosome of apical neural progenitor cells that is involved in mitotic spindle orientation during embryonic neurogenesis^27^ and is important for the correct proliferation and differentiation of neural progenitor cells during brain development.^28^ Mutations inthis gene cause autosomal recessive primary microcephaly 5, characterized by ID, microcephaly, sloping forehead, hypoplasia of the corpus callosum, simplified gyral pattern, and speech problems.^29,30^
WD repeat-containing protein 62; *WDR62*	*WDR62* is involved in spindle dynamics and organization, and is important for the proliferation of neural stem cells.^31,32^ Mutations in this gene cause autosomal recessive primary microcephaly 2, with or without cortical malformations. These patients show microcephaly, cortical malformations, developmental delays, and seizures.^33^
Cys2His2 zinc finger (C2H2-ZNF); *ZNF335*, ZNF526, *ZNF804A*	C2H2 zinc-finger proteins are the largest family of human TFs. They play a critical role in the transcriptional regulation of neural stem cells that rise to neurons and glial cells; therefore, proper function of these TFs is crucial for normal brain development.^34^
Exosome complex (EXOSC); *EXOSC2*, *EXOSC3*, *EXOSC5*	The EXOSC gene family includes genes responsible for the formation of the RNA-exosome complex. This complex is vital to RNA processing. It consists of ten conserved subunits, including EXOSC1-3 as non-catalytic cap components, EXOSC4-9 as a non-catalytic core, and DIS3 with both exoribonuclease and endonuclease activity.^35-37^ Studies on zebrafish have suggested that loss of EXOSC2 would lead to reduced small size; loss of spinal motor neurons and disturbance in EXOSC3 would result in reduced brain size and defects in the development of spinal motor neurons and the cerebellum.^38,39^ Loss of function of EXOSC5 in zebrafish causes reduced head and eye size as well as edema.^40^
General transcription factor IID complex subunits (TAF); *TAF1*, *TAF2*, *TAF6*	General TFIID is essential for the transcription initiation of RNA polymerase II. TFIID is a complex consisting of a TBP and 13 conserved factors called TAFs.^41,42^ *TAF1* encodes the largest subunit of TFIID, and is involved in early brain development. RT-PCR studies on cells harboring loss of *TAF1* showed changes in gene expression of neuronal ion channels.^43^ *TAF2* acts as a stabilizer in binding TFIID to the core promoter.^44^ *TAF6 *encodes part of the core of the TFIID complex, and defective *TAF6* can alter the assembly of TFIID.^45^
Vacuolar Protein Sorting 13 Homolog B; *VPS13B*	This gene encodes a protein that is important for non-vesicular lipid transport through intracellular membrane contact sites, and disorganizations in lipid constituents of organelle membranes would cause neurological disorders.^46^ Studies on flies also showed that *VPS13B* is necessary for the homeostasis of brain proteins.^47^ Mutations in this gene would result in a well-characterized disorder, Cohen syndrome, with common clinical features, including ID, developmental delay, microcephaly, eye problems, and facial characteristics.^48^
Steroid 5-alpha reductase family (SRD5A); *SRD5A3*	This gene encodes an enzyme called steroid 5a-reductase type 3, which is vital for N-glycosylation in the endoplasmic reticulum and has a crucial role in catalyzing the conversion of polyprenol to dolichol.^49,50^ Mutation in this gene causes Kahrizi syndrome with ID, cataracts, coloboma, kyphosis, and coarse facial features in our cohort.^51^
La Ribonucleoprotein 7 transcriptional regulator;* LARP7*	This gene encodes a transcriptional regulator protein that acts by binding to 7SK RNA and acts as an inhibitor of transcription by RNA polymerase II.^52^ Knockdown experiments on rats showed that inhibition of *LARP7* could inhibit protein synthesis and reduce ribosomes in hippocampal neurons.^53^ Mutations in this gene cause LARP7 deficiency, characterized by ID, developmental delay, skeletal anomalies, and behavioral problems.^54^
Calpains (CAPN); *CAPN10*, *CAPN9*	Calpains are a highly conserved group of calcium-dependent cysteine proteases that regulate synaptic plasticity and programmed neuronal death.^55,56^ They are essential for early embryo development through nuclear factor kappa-light-chain-enhancer of activated B cells (NF-κB) and Wingless-related integration site (Wnt) pathways.^57^
tRNA methyltransferases (TRMT); *TRMT1*, *TRMT10A*	Both genes encode tRNA methyltransferases that are involved in various cellular functions. Studies have shown that *TRMT10A* is highly expressed in the embryonic and fetal brain^58^ and defective *TRMT1* can enhance redox homeostasis. As a result, neural stem cells deteriorate due to higher sensitivity to reactive oxygen species and perturb normal neurogenesis.^59^
DEAD-box helicases (DDX); *DDX3X*, *DDX50*	The DEAD-box helicase family is a large family of ATP-dependent RNA helicases with a highly conserved Asp-Glu-Ala-Asp [D-E-A-D] motif that is involved in RNA metabolism.^60^ Studies have shown that alterations in *DDX3X* would lead to perturbation of RNA metabolism and alter the development of the brain cortical region.^61^
Kinesins (KIF); *KIF7*, *KIF11*, *KIF4A*	Kinesins are evolutionarily conserved motor proteins, important for the development of the brain and nervous system. They are involved in various biological functions, including cell division and intracellular trafficking.^62^
L-2-hydroxyglutarate dehydrogenase; *L2HGDH*	This gene provides L-2-hydroxyglutarate dehydrogenase, a mitochondrial enzyme involved in the conversion of L-2-hydroxyglutarate to 2-ketoglutarate.^63^ Studies in mice have shown that a defective form of L-2-hydroxyglutarate dehydrogenase leads to white matter abnormalities, neuroinflammation, improper neurogenesis of the hippocampal region, and neurodegeneration.^64^ Mutations in this gene cause L-2-hydroxyglutaric aciduria, characterized by ID, cerebellar ataxia, epilepsy, speech problems, and an increased amount of L-2-hydroxyglutaric acid in urine, blood, and cerebrospinal fluid.^63^
Lines Homolog 1; *LINS1*	Mutations in *LINS1 *deteriorate the proper function of the WNT signaling pathway, which is involved in the development of the central nervous system and affects cell fate determination in neuronal progenitor cells, neuronal migration and polarization, and synaptogenesis.^65,66^ Mutations in* LINS1* lead to intellectual developmental disorder, autosomal recessive 27.
Transmembrane Protein 67; *TMEM67*	*TMEM67* encodes Meckelin, a transmembrane protein involved in cerebellar development that controls the Wnt/β-catenin signaling pathway.^67^ During development and differentiation, Meckelin can act as a WNT receptor and is also involved in centrosome migration during ciliogenesis and primary cilium formation.^68^ Mutations in *TMEM67* can cause a variety of ciliopathies, including Meckel syndrome, Joubert syndrome, and COACH syndrome 1.^68^ Here, we report three families with a mutation in *TMEM67* that caused Joubert syndrome 6, which is categorized with ID, hypoplasia of the cerebellar vermis, molar tooth sign, hypotonia, developmental delay, ataxia, and renal problems.
BBSome; *BBS7*,* BBS9*,* BBS4*	BBSome is an octameric complex involved in protein trafficking of the ciliary membrane and non-ciliary functions, including the localization of receptors in the cell membrane.^69,70^ This complex is essential for the appropriate functioning of astrocytes in the brain. Studies have shown that disruption of BBSome causes defects in primary cilia and affects the morphology and metabolism of neurons in the brain.^71,72^ Mutations in the subunits of the BBSome complex cause Bardet-Biedl syndrome, categorized with ID, central obesity, hypogonadism, retinal dystrophy, renal problems, and post-axial polydactyly.^73,74^

TFs, transcription factors; ID, Intellectual disability; TFIID, transcription factor IID; TBP, TATA-binding protein; TAF, TBP-associated factor; RT-PCR, Real-time polymerase chain reaction.

**Table 2:** Details of the Families with Common ID Genes in our Cohort

**Genes and the Categories**		**Chromosomal Variant**^#^	**OMIM Phenotype**	**Mode of Inheritance**	**Ethnicity of the Families**
AP4 complex	*AP4B1*	NC_000001.10:g.114442649del^a^	614066	AR	Persian
		NC_000001.10:g.114441378_114441379del^a^			
		NC_000001.10:g.114441425T > C^a^			
	*AP4E1*	NC_000015.9:g.51242065_51242066insNN^c^	613744	AR	Azeri
	*AP4M1*	NC_000007.13:g.99701748G > A^c^	612936	AR	Kurd
		NC_000007.13:g.99703887A > C^b^			Persian
		NC_000007.13:g.99700491del^a^			Persian
		NC_000007.13:g.99703627G > A^a^			Turkmen
		NC_000007.13:g.99701748G > A^a^			Persian
		NC_000007.13:g.99701748G > A^a^			Persian
	*AP4S1*	NC_000014.8:g.31542174C > T^a^	614067	AR	Baluch
		NC_000014.8:g.31542174C > T^a^			Persian
ASPM		NC_000001.10:g.197111490_197111491del^b^	608716	AR	Baluch
		NC_000001.10:g.197070329_197070330dup^a^			Persian
		NC_000001.10:g.197070283G > A^a^			Azeri
		NC_000001.10:g.197091611_197091612del^a^			Persian
		NC_000001.10:g.197070599_197070600del^a^			Persian
		NC_000001.10:g.197115270C > G^d^ NC_000001.10:g.197091611_197091612del^d^			Persian
WDR62		NC_000019.9:g.36575602A > G^a^	604317	AR	Persian
		NC_000019.9:g.36546051G > T^a^			
		NC_000019.9:g.36594088_36594089del^a^			
		NC_000019.9:g.36582182C > T^c^			
		NC_000019.9:g.36594255del^d^			
		NC_000019.9:36558235G > A^d^			
C2H2-Zinc Finger	*ZNF335*	NC_000020.10:g.44588870G > A^a^	615095	AR	Persian
		NC_000020.10:g.44578005A > C^a^			Persian
	*ZNF526*	NC_000019.9:g.42730172G > C^c^	619877	AR	Baluch
		NC_000019.9:g.42729931G > A^c^			Kurd
	*ZNF804A*	NC_000002.11:g.185731147G > A^b^	ID	AR	Persian
Exosome complex	*EXOSC2*	NC_000009.11:g.133578439G > T^b^	617763	AR	Persian
		NC_000009.11:g.133578439G > T^d^			Persian
	*EXOSC3*	NC_000009.11:g.37783990T > G^d^	614678	AR	Persian
		NC_000009.11:g.37783990T > G^d^			Persian
	*EXOSC5*	NC_000019.9:g.41897789G > A^a^	619576	AR	Persian
General transcription factor IID complex subunits	*TAF1*	NC_000023.10:g.70588006C > G^b^	300966	XLR	Persian
		NC_000023.10:g.70607141A > G^a^			
	*TAF2*	NC_000008.10:g.120795788A > G^c^	615599	AR	Persian
		NC_000008.10:g.120805628C > A^d^			Kurd
	*TAF6*	NC_000007.13:g.99711522A > G^a^	617126	AR	Azeri
VPS13B		NC_000008.10:g.100732719del^a^	216550	AR	Persian
		NC_000008.10:g.100832347_100832380delinsC^a^			Persian
		NC_000008.10:g.100732719del^a^			Persian
		NC_000008.10:g.100832269del^a^			Arab
		NC_000008.10:g.100568867G > A^a^			Persian
Steroid 5-alpha reductase family	*SRD5A3*	NC_000004.11:g.56212560G > A^b^	612713	AR	Persian
		NC_000004.11:g.56230382A > G^c^			Baluch
		NC_000004.11:g.56212705_56212706insN^c^			Persian
		NC_000004.11:g.56212707dup^f^			Baluch
*LARP*7		NC_000004.11:g.113575316G > C^a^	615071	AR	Persian
		NC_000004.11:g.113568633C > T^g^			Persian
		NC_000004.11:g.113578402_113578405del^g^			Azeri
		NC_000004.11:g.113568536_113568537insN^c^			Turk
*Calpains*	*CAPN10*	NC_000002.11:g.241530371_241530376insN[15]^c^	601283	AR	Persian
		NC_000002.11:g.241528849T > A^a^			Arab
		NC_000002.11:g.241530301C > T^a^			Persian
	*CAPN9*	NC_000001.10:g.230898426G > T^a^	ID	AR	Arab
tRNA methyltransferases	*TRMT1*	NC_000019.9:g.13223781_13223812del^c^	618302	AR	Arab
		NC_000019.9:g.13223781_13223812del^a^			Baluch
		NC_000019.9:g.13220260_13220261del^a^			Azeri
	*TRMT10A*	NC_000004.11:g.100478552G > T^a^	616033	AR	Persian
DEAD-box helicases	*DDX3X*	NC_000023.10:g.41204441T > A^b^	300958	XLR	Persian
		NC_000023.10:g.41203594A > G^a^			
		NC_000023.10:g.41204491C > T^a^			
	*DDX50*	NC_000010.10:g.70706241_70706264del^b^	ID	AR	
Kinesins	*KIF11*	NC_000010.10:g.94366083C > T^b^	152950	AD	Persian
	*KIF4A*	NC_000023.10:g.69607097C > T^a^	300923	XLR	Turk
	*KIF7*	NC_000015.9:g.90185556C > T^c^	200990	AR	Persian
		NC_000015.9:g.90195903T > C^b^			Arab
L-2-hydroxyglutarate dehydrogenase	*L2HGDH*	NC_000014.8:g.50750723G > A^a^	236792	AR	Persian
		NC_000014.8:g.50734532G > A^c^			Persian
		NC_000014.8:g.50768804A > T^d^			Lur
*LINS*1		NC_000015.9:g.101114094_101114097del^a^	614340	AR	Persian
		NC_000015.9:g.101120983del^a^			Kurd
		NC_000015.9:g.101114094_101114097del^c^			Persian
*TMEM6*7		NC_000008.10:g.94792831A > G^b^	610688	AR	Persian
		NC_000008.10:g.94792831A > G^a^			
		NC_000008.10:g.94792831A > G^a^			
BBSome	*BBS4*	NC_000015.9:g.73002041_73004648del^a^	615982	AR	Persian
	*BBS7*	NC_000004.11:g.122754467_122754472del^c^	615984	AR	
	*BBS9*	NC_000007.13:g.33397608G > A^a^	615986	AR	

ID, intellectual disability; AR, autosomal recessive; XLR, X-linked recessive; AD, autosomal dominant; NA, not assigned. ^#^ Based on GRCh37(hg19). ^a^ These families were first reported in our previous study.^1^ ^b^ These families were first reported in our previous study.^8^ ^c^ These families were first reported in our previous study.^9^ ^d^ These families were first reported in our previous study.^35^ ^e^ Unpublished data. ^f^ The family was first described in a previous paper.^75^ ^g^ These families were first reported in our previous study.^10^

**Table 3:** Genes with a Mutation in Two Unrelated Affected Families

**Gene**	**Chromosomal variants**^#^	**OMIM phenotype**	**Ethnicity of the families**
*ADGRG1*	NC_000016.9:g.57695619C > T^b^	606854	Persian
	NC_000016.9:g.57695794G > A^a^		
*AHI1*	NC_000006.11:g.135778798G > A^c^	608629	Persian
	NC_000006.11:g.135769570C > T^c^		
*AIMP1*	NC_000004.11:g.107258194G > C^b^	260600	Persian
	NC_000004.11:g.107252964T > G^a^		
*AK1*	NC_000009.11:g.130630703C > T^a^	612631	Persian
	NC_000009.11:g.130634140G > A^a^		Arab
*ALS2*	NC_000002.11:g.202569196A > G^a^	205100	Persian
	NC_000002.11:g.202619225C > T^a^		Arab
*ASNS*	NC_000007.13:g.97488183A > C^b^	615574	Persian
	NC_000007.13:g.97498245T > C^a^		
*ATP8A2*	NC_000013.10:g.26125642G > T^b^	615268	Persian
	NC_000013.10:g.26436510G > A^b^		
*ATRX*	NC_000023.10:g.76855934A > G^a^	309580	Persian
	NC_000023.10:g.76875953C > G^a^		
*B3GALNT2*	NC_000001.10:g.235643447G > A^a^	615181	Azeri
	NC_000001.10:g.235621957C > T^a^		Persian
*CASK*	NC_000023.10:g.41416344G > C^a^	300422	Arab
	NC_000023.10:g.41519706G > A^a^		Persian
*CDK5RAP2*	NC_000009.11:g.123201968_123201971del^b^	604804	Persian
	NC_000009.11:g.123253590_123253593del^a^		Baluch
*CEP104*	NC_000001.10:g.3742330_3742331insAA^a^	616781	Persian
	NC_000001.10:g.3746500dup^a^		Lur
*DYM*	NC_000018.9:g.46889551del^a^	223800	Persian
	NC_000018.9:g.46808420G > A^a^		
*ELP2*	NC_000018.9:g.33736538G > T^c^	617270	Azeri
	NC_000018.9:g.33739953A > C^c^		Turk
*ERLIN2*	NC_000008.10:g.37599315_37599677delinsCTGTG^a^	611225	Azeri
	NC_000008.10:g.37595547G > A ^c^		Persian
*GAMT*	NC_000019.9:g.1398999del^a^	612736	Persian
	NC_000019.9:g.1398999del^a^		
*GMPPA*	NC_000002.11:g.220368858C > A^b^	615510	Persian
	NC_000002.11:g.220370723G > A^a^		
*IPP*	NC_000001.10:g.46179920_46185014del^a^	ID	Persian
	NC_000001.10:g.46182687C > T^a^		
*ITGAV*	NC_000002.11:g.187541960_187541962del^a^	ID	Persian
	NC_000002.11:g.187529348G > A^a^		Arab
*MAN1B1*	NC_000009.11:g.139995540C > T^c^	614202	Persian
	NC_000009.11:g.140001735del^d^		Lur
*NDST1*	NC_000005.9:g.149922489G > T^a^	616116	Persian
	NC_000005.9:g.149925029G > A^c^		
*NEURL4*	NC_000017.10:g.7224505G > A^b^	ID	Persian
	NC_000017.10:g.7222392dup^a^		
*ORC1*	NC_000001.10:g.52851591G > A^a^	224690	Lur
	NC_000001.10:g.52850232T > C^a^		Turkmen
*PIDD1*	NC_000011.9:g.800015C > T^a^	ID	Persian
	NC_000011.9:g.799846G > A^a^		
*PRKCG*	NC_000019.9:g.54394928_54396645del ^c^	605361	Persian
	NC_000019.9:g.54403866G > T^c^		
*PRRT2*	NC_000016.9:g.29825024dup^a^	602066	Kurd
	NC_000016.9:g.29825015_29825016insN^c^		
*RDH11*	NC_000014.8:g.68145040dup^a^	616108	Persian
	NC_000014.8:g.68159744T > C^d^		Persian
*RNASEH2C*	NC_000011.9:g.65487533G > A^b^	610329	Persian
	NC_000011.9:g.65487856G > A^a^		Baluch
*RNFT2*	NC_000012.11:g.117274037T > C^a^	ID	Persian
	NC_000012.11:g.117274037T > C^a^		
*SCAPER*	NC_000015.9:g.77064235G > A^a^	618195	Baluch
	NC_000015.9:g.77064240_77064241insN^c^		Persian
*SUCLA2*	NC_000013.10:g.48528645G > A^b^	612073	Kurd
	NC_000013.10:g.48562777T > G^b^		Azeri
*SURF1*	NC_000009.11:g.136219373A > G^c^	220110	Turk
	NC_000009.11:g.136218979C > A^a^		Arab
*TSEN54*	NC_000017.10:g.73513639G > T^b^	610204	Persian
	NC_000017.10:g.73513639G > T^a^		Kurd
*TTC5*	NC_000014.8:g.20766998del^a^	619244	Turk
	NC_000014.8:g.20774045C > T^a^		Baluch
*TWN*K	NC_000010.10:g.102748841C > A^a^	616138	Persian
	NC_000010.10:g.102748841C > A^d^		Persian
*UBE3B*	NC_000012.11:g.109921396G > A^b^	244450	Persian
	NC_000012.11:g.109935697T > C^a^		Arab

ID, intellectual disability. ^#^ Based on GRCh37(hg19). ^a^ These families were first reported in our previous study.^1^ ^b^ These families were first reported in our previous study.^8^ ^c^ These families were first reported in our previous study.^9^ ^d^ Unpublished data.

###  Comparison of our Study with Seven Studies Reporting ID Cohorts

**Figure 3:**
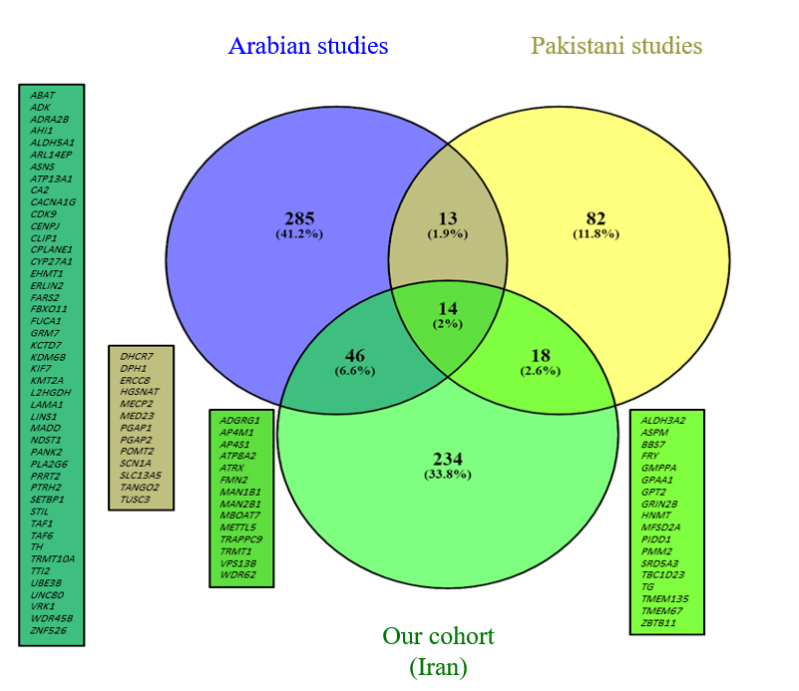
Venn Diagram Showing ID Genes Reported in our Cohort and the Pakistani and Arab Groups.

**Figure 4:**
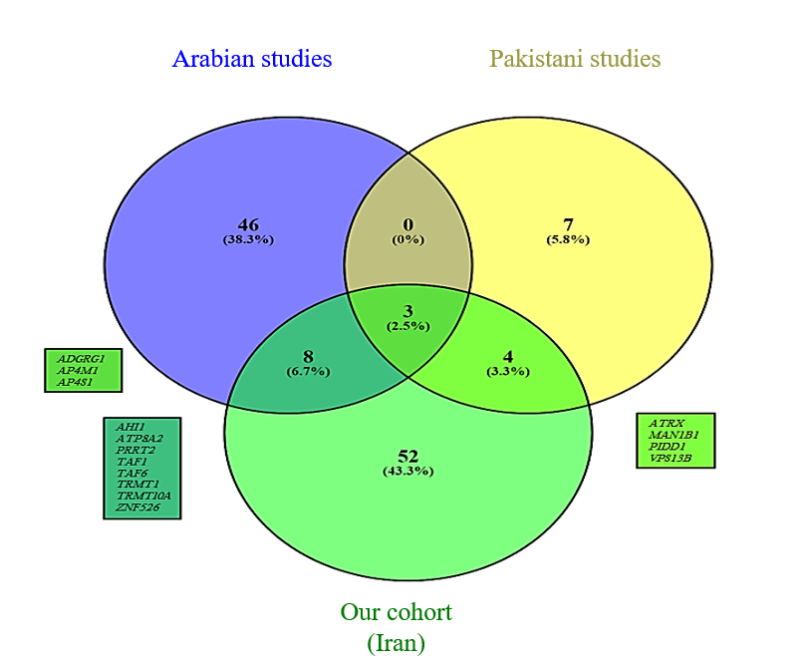
Venn Diagram Showing Repetitive ID Genes Reported in our Cohort and the Pakistani and Arab Groups.

 We compiled two lists of reported ID genes among seven studies from neighboring countries with a high consanguinity rate that included ID cohorts.^12-18^ This comparison resulted in the Venn diagram depicted in Figure 3. We also extracted repetitive genes (Supplementary File 1, Table S1) embedded in these three lists and compared them by depicting another Venn diagram shown in Figure 4.^76^ The details of these comparisons are shown in Table 4. For both of these comparisons, copy number variations were excluded.

**Table 4:** Details of Gene Comparison Between Pakistani and Arab Groups

**Cohorts/Number of Genes**	**Our Cohort (Iranian Population)**	**Pakistani Groups**	**Arab Groups**
Total number of genes	312	127	358
Number of repetitive genes in total	67	14	57

## Discussion

 Based on an epidemiological study of a large Iranian ID cohort, we were able to categorize the most common ID genes into 17 groups (AP4 complex, *ASPM*, *WDR62*, C2H2-Zinc fingers, exosome complex genes, General transcription factor IID subunits, *VPS13B*, *SRD5A3*, *LARP7*, calpain genes, tRNA methyltransferases, kinesins, DEAD-box helicases, *L2HGDH*, *LINS1*, *TMEM67*, and BBSome complex genes). Each group was repeatedly reported for at least three families in our cohort. Because of the high consanguinity rate in our population, 87.87% of these genes demonstrated an AR mode of inheritance. The most common syndromic ID in our study was AP4 deficiency syndrome, which was reported in 12 families and the most common non-syndromic ARID gene was *ASPM*.

 For 36 ID genes, we could identify two unrelated families. For several genes, we found two unrelated families with the same mutations. These included families with (NC_000008.10:g.100732719del, p.Phe2293Leufs*24) in *VPS13B*, families with (NC_000012.11:g.117274037T > C, p.Cys384Arg) in *RNFT2*,and families with (NC_000019.9:g.1398999del, p.Gly164Alafs*14) in *GAMT*. In another study in 2015, Rafiq et al reported (p.Phe2293Leufs*24) in two unrelated Pakistani families of Baloch population.^77^ On the other hand, for the recurrent variant in *TMEM67*, Dehghani et al found the same mutation among 12 Iranian nuclear families and suggested the variant as a founder mutation in the Iranian population.^78^ Our study supports this hypothesis and confirms the prioritization of this variant for the diagnosis of Iranian patients with Joubert syndrome. At the same time, more studies are needed to confirm our hypothesis. Studies have shown that the variant of *GAMT* has been reported frequently in various families from Turkey, Israeli Arabs, Italy, and Iran.^1,79-81^ It seems that the glycine at position 164 is a highly conserved amino acid, and a mutation at this position is one of the most prevalent alterations in *GAMT*.

 According to HGMD and ClinVar, worldwide epidemiological studies on ARID showed that only a small number of these genes appear to have frequent variant reports, including *GALT*, *VPS13B*, *ASPM*, *SPG11*, *MUT*, *GLDC*, *CEP290*, *POLG*, *LAMA2*, and *SMPD1*.^6^ Two of these genes (*VPS13B*, *ASPM*) were also frequent in our cohort. In 2018, Jamra^6^ estimated that because both these syndromic genes have been well-known for a long time, a large number of reports are available. Although these genes have been known for a long time, our cross-sectional data showed a high prevalence of both genes, suggesting that they are two prevalent ARID genes.

 The comparison of ID genes between our Iranian cohort, the Pakistani cohort, and Arab cohorts showed that Iran and Arabs have more common genes in comparison to Pakistani cohort. At this stage, we cannot claim that this similarity in ID genes is due to a more similar genetic background between these two groups of people, and more comprehensive studies are needed. We found 14 genes common between the three groups including *ADGRG1*, *AP4M1*, *AP4S1*, *ATP8A2*, *ATRX*, *FMN2*, *MAN1B1*, *MAN2B1*, *MBOAT7*, *METTL5*, *TRAPPC9*, *TRMT1*, *VPS13B*, and *WDR62*. The first three genes (*AP4M1* and *AP4S1* cause AP4 deficiency syndrome and *ADGRG1* causes bilateral frontoparietal polymicrogyria) are repeated among these three groups of people, and they seem to be among the most common ID genes in consanguineous marriages.

 Along with much better recognition of the role of genetic factors in ID in recent decades, the gap in epidemiological studies of genetic factors in ID has become more evident, and a large number of genes involved in this phenotype are yet to be discovered. Defining the prevalence of ID-mutated genes in Iran and having accurate statistical data help us make better strategic decisions on genetic and clinical diagnostics of IDs in the Iranian population and prevent the occurrence of such costly disabilities. Due to the large sample size, our data could enhance the design of targeted NGS platforms, mainly population-specific diagnostic tools.

## Supplementary Files

Supplementary file 1 contains Table S1.
